# Supplementary material for: RNA Pol IV induces antagonistic parent-of-origin effects on Arabidopsis endosperm
Source: PLoS Biol. 2022 Apr 7;20(4):e3001602. doi: 10.1371/journal.pbio.3001602 (PMC9017945; doi:10.1371/journal.pbio.3001602)
Supplement: S1 Fig — Loss of maternal NRPD1 decreases paternal excess seed viability while loss of paternal NRPD1 increases seed viability. Each dot in the aligned dot plot represents seed viability from one paternal excess cross (biological replicate). Significance of difference between indicated crosses was calculated by Wilcox test. Underlying data can be found in S1 Data. Pol IV, polymerase IV. (PDF) [file pbio.3001602.s001.pdf]

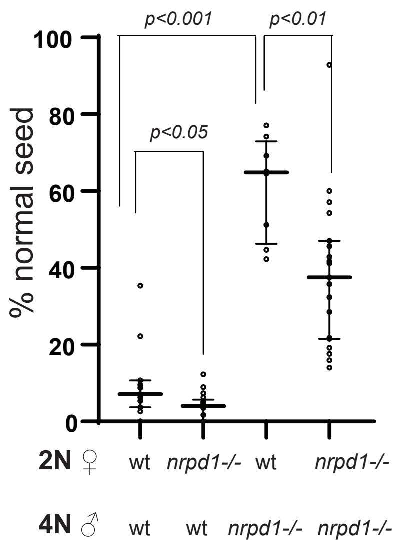

S1 Fig. Maternal and paternal Pol IV activity have opposing effects on seed abortion caused by paternal genomic excess. Loss of maternal *NRPD1* decreases paternal excess seed viability while loss of paternal *NRPD1* increases seed viability. Each dot in the aligned dot plot represents seed viability from one paternal excess cross (biological replicate). Signficance of difference between indicated crosses was calculated by Wilcox test. Underlying data can be found in S1 Data.
