# Supplementary material for: RNA Pol IV induces antagonistic parent-of-origin effects on Arabidopsis endosperm
Source: PLoS Biol. 2022 Apr 7;20(4):e3001602. doi: 10.1371/journal.pbio.3001602 (PMC9017945; doi:10.1371/journal.pbio.3001602)
Supplement: S2 Fig — (A) Size (nt) of all sRNAs in endosperm sRNA peaks predominated by 21, 22, 23, or 24-nt sRNAs. ShortStack was used to call peaks in WT (Ler × Col-0) endosperm. Each peak is grouped into a size class based on the predominant size of the sRNA species in that peak. Fraction of sRNAs at other sizes in the same peaks are plotted. (B) sRNA peaks of multiple sizes are impacted by loss of NRPD1. (C) Upset plot shows that genes losing sRNAs of one size class lose sRNAs of other size classes in nrpd1−/− endosperm. Data for S2A and S2B Fig can be found in S1 Data. Gene lists used for upset graph in S1C Fig can be extracted from GEO GSE197717. Pol IV, polymerase IV; sRNA, small RNA; WT, wild type. (PDF) [file pbio.3001602.s002.pdf]

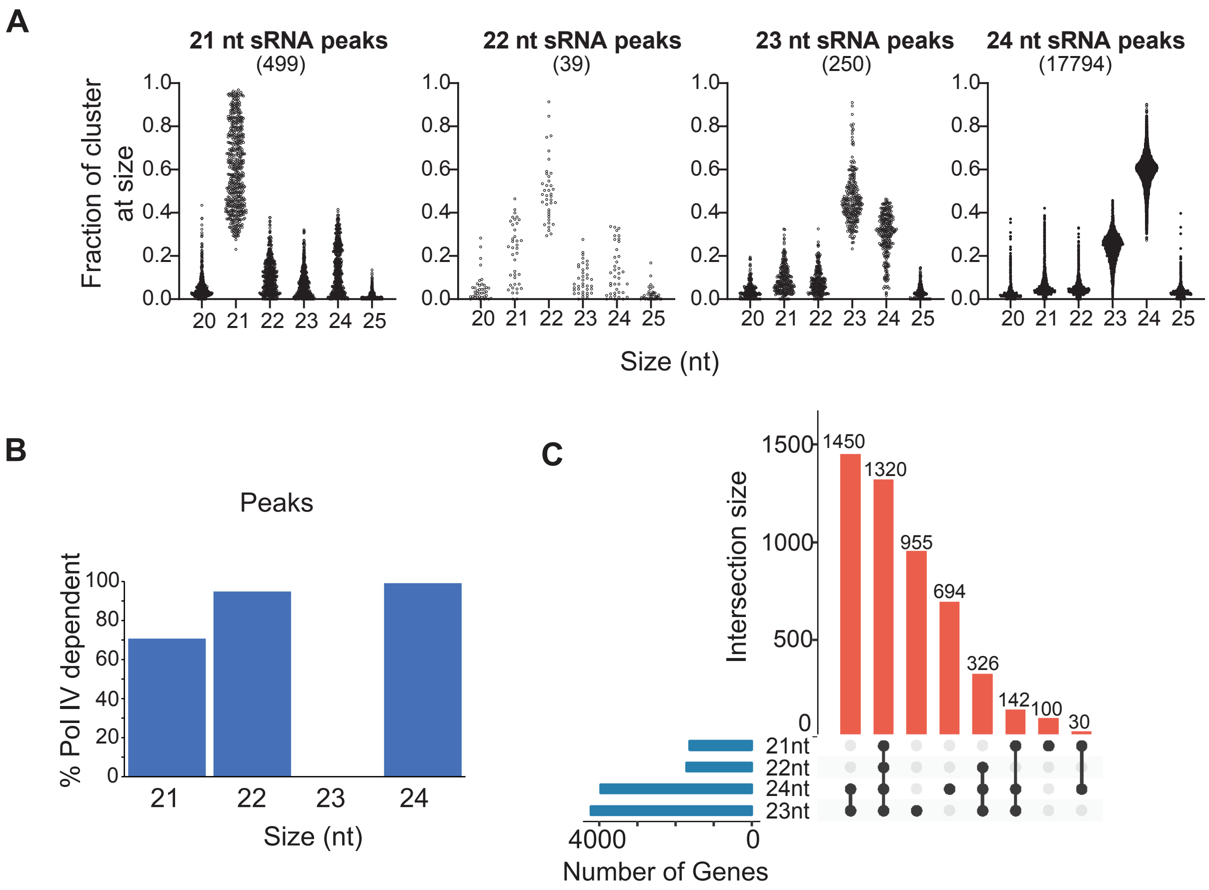

S2 Fig. RNA Pol IV is necessary for the production of 21-24 nt sRNAs in the endosperm.
(A) Size (nt) of all sRNAs in endosperm small RNA peaks predominated by 21, 22, 23, or 24 nt sRNAs. ShortStack was used to call peaks in wild-type (L*er* x Col-0) endosperm. Each peak is grouped into a size class based on the predominant size of the small RNA species in that cluster. Fraction of small RNAs at other sizes in the same peaks are plotted. **(B)** Small RNA peaks of multiple sizes are impacted by loss of *NRPD1*. **(C)** Upset plot shows that genes losing sRNAs of one size class lose sRNAs of other size classes in *nrpd1-/-* endosperm. Data for S2A-B Fig can be found in S1 Data. Gene lists used for upset graph in S1C Fig can be extracted from GEO GSE197717.
