# Supplementary material for: RNA Pol IV induces antagonistic parent-of-origin effects on Arabidopsis endosperm
Source: PLoS Biol. 2022 Apr 7;20(4):e3001602. doi: 10.1371/journal.pbio.3001602 (PMC9017945; doi:10.1371/journal.pbio.3001602)
Supplement: S3 Fig — (A) One parent’s copy of NRPD1 is sufficient for 24-nt sRNA production from genes and TEs at most loci, here exemplified by RIC5 and a VANDAL21 insertion. (B) Examination of 21- to 24-nt sRNAs over genes and TEs shows that inheriting a maternal mutation in NRPD1 has a greater impact than inheriting a paternal mutation in NRPD1. Loci with differential sRNA expression were identified using DESeq2. WT read counts represent average read counts per locus across 3 replicates. Reads mapping to TE insertions were normalized using genic sRNA expression. Black circles represent padj ≤ 0.05. Gray circles represent padj > 0.05. Data for this plot can be found in S2 Data. sRNA, small RNA; TE, transposable element; WT, wild type. (PDF) [file pbio.3001602.s003.pdf]

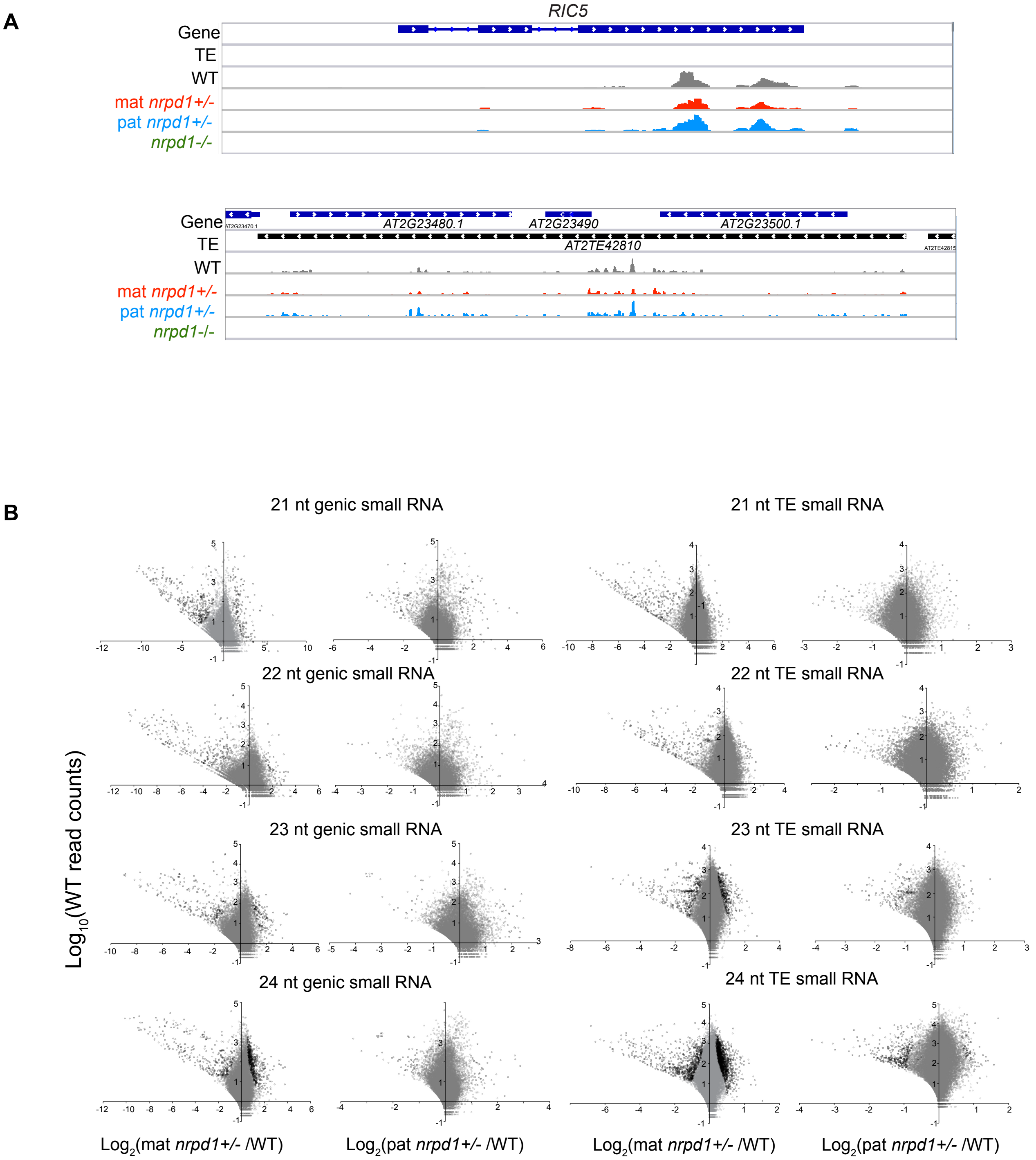

S3 Fig. Impact of the loss of maternal and paternal *NRPD1* on endosperm small RNA populations.
(A) One parent's copy of *NRPD1* is sufficient for 24 nt sRNA production from genes and TEs at most loci, here exemplified by *RIC5* and a VANDAL21 insertion. **(B)** Examination of 21-24 nt sRNAs over genes and TEs shows that inheriting a maternal mutation in *NRPD1* has a greater impact than inheriting a paternal mutation in *NRPD1*. Loci with differential sRNA expression were identified using DESeq2. Wild-type (WT) read counts represent average read counts per locus across three replicates. Reads mapping to TE insertions were normalized using genic sRNA expression. Black circles represent *padj≤0.05*. Gray circles represent *padj>0.05*. Data for this plot can be found in S2 Data.
