# Supplementary material for: RNA Pol IV induces antagonistic parent-of-origin effects on Arabidopsis endosperm
Source: PLoS Biol. 2022 Apr 7;20(4):e3001602. doi: 10.1371/journal.pbio.3001602 (PMC9017945; doi:10.1371/journal.pbio.3001602)
Supplement: S4 Fig — SNPs between Col-0 and Ler were used to identify the parental origins of sRNAs arising from genes and TEs. Differentially expressed loci were identified using DESeq2 as described in Fig 1. Loci with a sum of at least 10 allele-specific reads in 3 WT Ler × Col-0 (WT) replicates and showing significant differences in 21-nt and 24-nt sRNAs in Ler nrpd1 −/− × Col nrpd1−/− endosperm were included. Box plots are Tukey plots. Numbers over box plots indicate the number of loci evaluated. Data represented in this figure can be found in S3 Data. Pol IV, polymerase IV; sRNA, small RNA; WT, wild type. (PDF) [file pbio.3001602.s004.pdf]

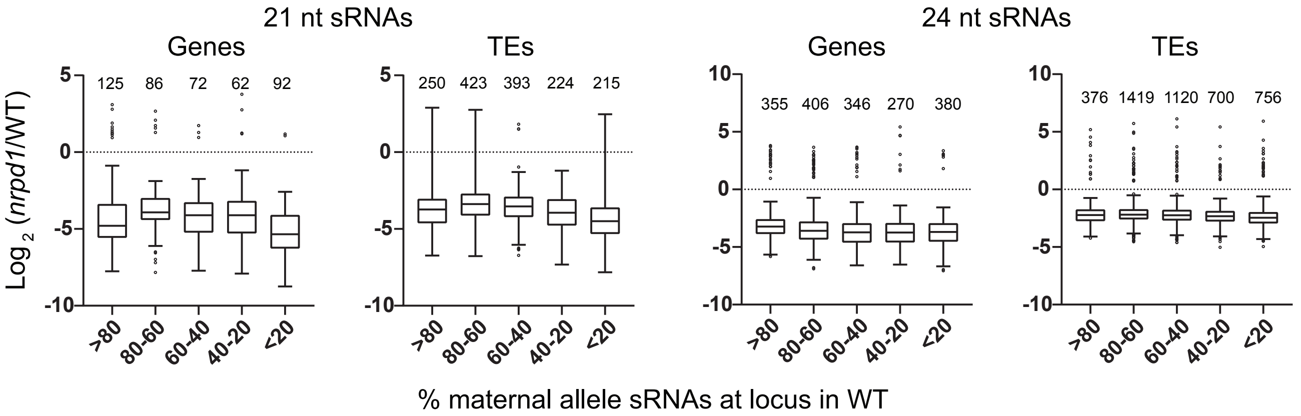


**S4 Fig. RNA Pol IV-dependent small RNAs arise from both maternal and paternal alleles**.
SNPs between Col-0 and L*er* were used to identify the parental origins of small RNAs arising from genes and TEs. Differentially expressed loci were identified using DESeq2 as described in Fig 1. Loci with a sum of at least ten allele-specific reads in three wild-type L*er* x Col-0 (WT) replicates and showing significant differences in 21 nt and 24 nt sRNAs in L*er* *nrpd1* -/- x Col *nrpd1*-/- endosperm were included. Box plots are Tukey plots. Numbers over box plots indicate the number of loci evaluated. Data represented in this figure can be found in S3 Data.
