# Supplementary material for: RNA Pol IV induces antagonistic parent-of-origin effects on Arabidopsis endosperm
Source: PLoS Biol. 2022 Apr 7;20(4):e3001602. doi: 10.1371/journal.pbio.3001602 (PMC9017945; doi:10.1371/journal.pbio.3001602)
Supplement: S5 Fig — For each mRNA-seq library built with RNA from dissected endosperm, reads overlapping genic loci were counted with Htseq-count. Enrichment of a seed tissue in each sample was then calculated using the tissue enrichment tool [68]. mRNA-seq, mRNA sequencing. (PDF) [file pbio.3001602.s005.pdf]

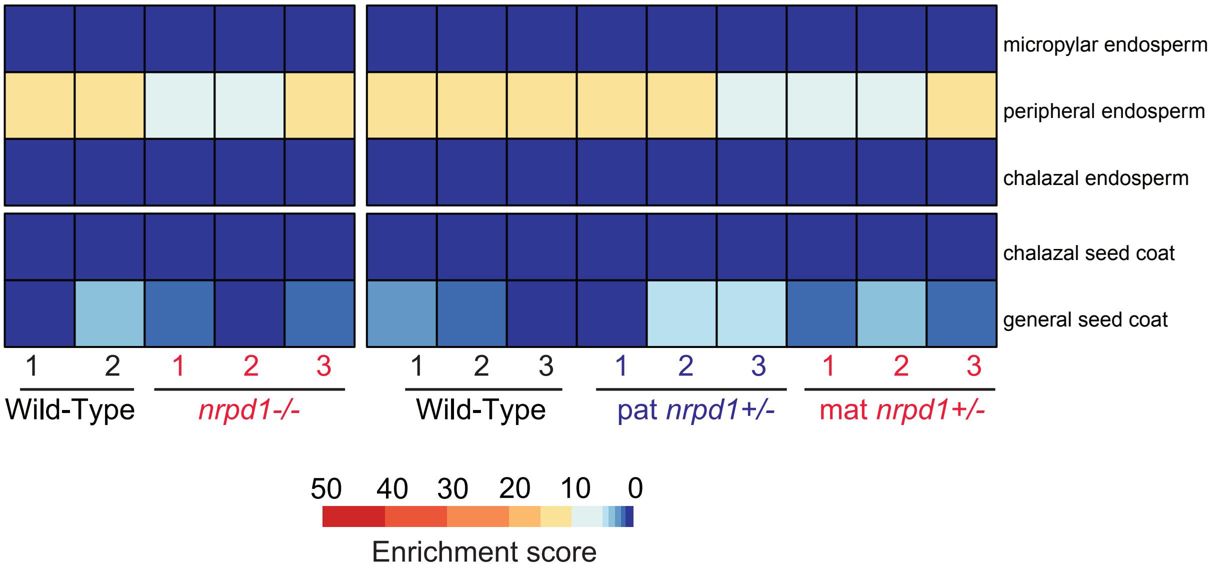

S5 Fig. Tissue enrichment in dissected endosperm shows little seed coat contamination.
For each mRNA-Seq library built with RNA from dissected endosperm, reads overlapping genic loci were counted with Htseq-count. Enrichment of a seed tissue in each sample was then calculated using the tissue enrichment tool [68].
