# Supplementary material for: RNA Pol IV induces antagonistic parent-of-origin effects on Arabidopsis endosperm
Source: PLoS Biol. 2022 Apr 7;20(4):e3001602. doi: 10.1371/journal.pbio.3001602 (PMC9017945; doi:10.1371/journal.pbio.3001602)
Supplement: S6 Fig — (A) A subset of imprinted genes are misregulated by loss of maternal or all NRPD1. Loss of paternal NRPD1 has limited impact on expression. Scatter plots show output from Cuffdiff calculating the difference in gene expression between WT and indicated mutant genotype. Black circles represent genes whose abundance varies by 2-fold and q < 0.05. All other genes represented by gray circles. (B) Aligned dot plot representing fold change for imprinted genes exhibiting significant differences in expression. (C) Allele-specific expression is not impacted at most imprinted loci. % maternal of all Col-Ler imprinted genes identified in [21] was calculated by counting reads overlapping Col/Ler SNPs. (D) Examples of imprinted genes whose allelic bias was impacted by loss of all NRPD1. In WT, WOX8 and SAC2 are predominantly expressed from maternal and paternal alleles, respectively. In nrpd1−/−, WOX8 is down-regulated because of reduced expression from the maternal allele, while the expression of SAC2 is driven by down-regulation of the paternal allele. Data represented in this figure can be found in S5 Data. Pol IV, polymerase IV; WT, wild type. (PDF) [file pbio.3001602.s006.pdf]

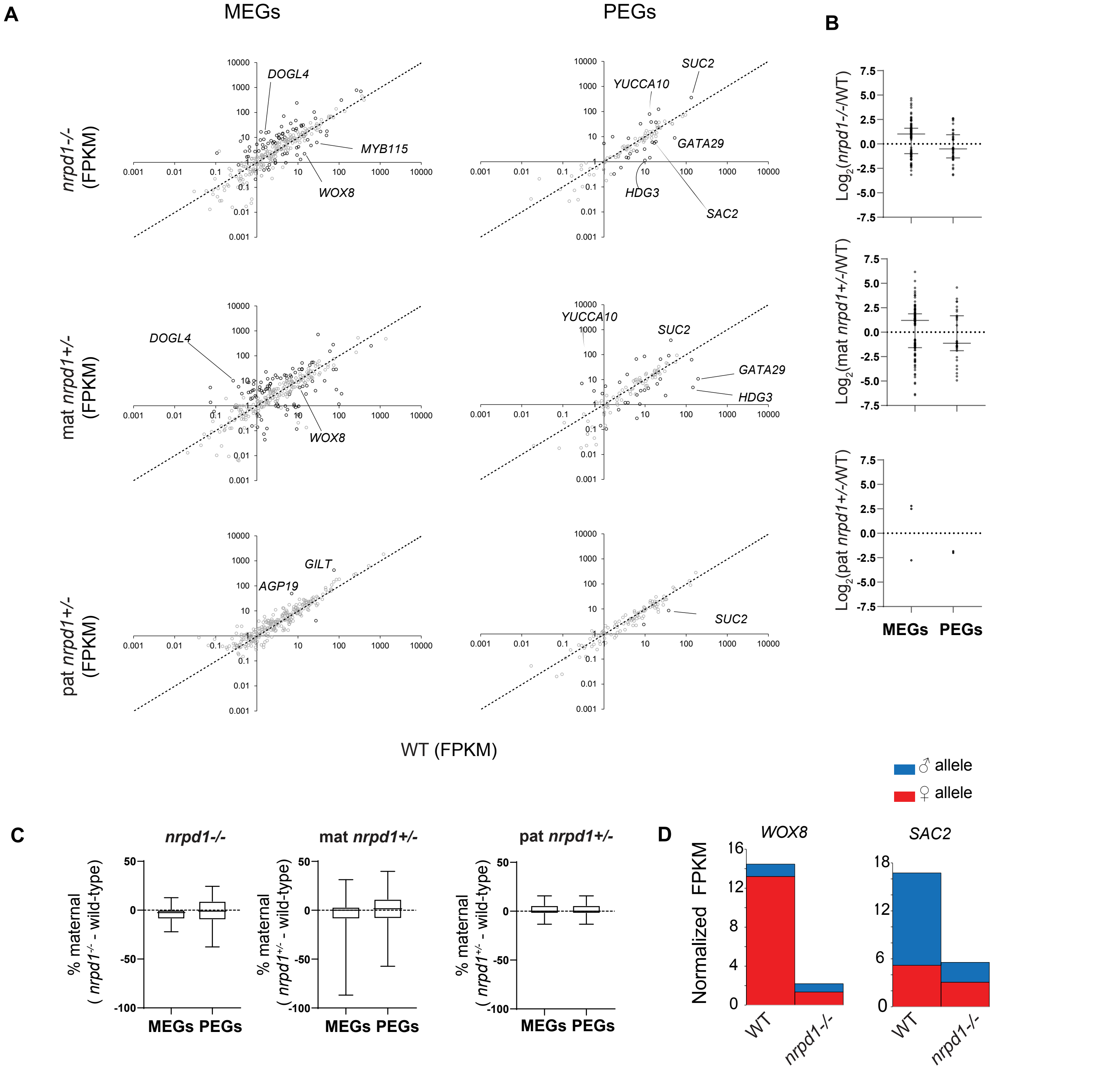

S6 Fig. Impact of Pol IV on imprinted gene expression and imprinting.

(A) A subset of imprinted genes are mis-regulated upon loss of maternal or all *NRPD1*. Loss of paternal *NRPD1* has limited impact on expression. Scatter plots show output from Cuffdiff calculating the difference in gene expression between WT and indicated mutant genotype. Black circles represent genes whose abundance varies by two-fold and *q≤0.05*. All other genes represented by gray circles. **(B)** Aligned dot plot representing fold-change for imprinted genes exhibiting significant differences in expression. **(C)** Allele-specific expression is not impacted at most imprinted loci. % maternal of all Col-L*er* imprinted genes identified in [21] was calculated by counting reads overlapping Col/L*er* SNPs. **(D)** Examples of imprinted genes whose allelic bias was impacted by loss of all *NRPD1*. In wild-type, *WOX8* and *SAC2* are predominantly expressed from maternal and paternal alleles, respectively. In *nrpd1-/-*, *WOX8* is down-regulated because of reduced expression from the maternal allele while the expression of *SAC2* is driven by down-regulation of the paternal allele. Data represented in this figure can be found in S5 Data.
