# Supplementary material for: RNA Pol IV induces antagonistic parent-of-origin effects on Arabidopsis endosperm
Source: PLoS Biol. 2022 Apr 7;20(4):e3001602. doi: 10.1371/journal.pbio.3001602 (PMC9017945; doi:10.1371/journal.pbio.3001602)
Supplement: S7 Fig — (A–C) Comparisons of genes showing significant differences in 21-, 22-, 24-nt sRNA and mRNA abundance shows that only a subset of genes (lower right quadrant) may be repressed by Pol IV–dependent sRNAs in WT. Differences in sRNA abundance between WT and nrpd1−/− were calculated using DESeq2. Differences in mRNA was calculated using Cuffdiff. Numbers in bold in each quadrant indicate number of genes. (D) NanoPARE data maps 5′ ends of transcripts and identifies TSSs and cleavage sites within the gene body. Change in mRNA cleavage at genes that show increased mRNA abundance and decreased 21-, 22-, or 24-nt sRNAs. Coverage of 5′ end reads from NanoPARE sequencing was calculated for every nucleotide in the genome. Difference in 5′ read coverage at each nucleotide was calculated for 2 replicates of WT endosperm (Ler × Col) and 3 nrpd1−/− (Ler nrpd1−/− × Col nrpd1−/−) replicates using DESeq2. Each point plotted on the dot plot represents one nucleotide with differential 5′ reads overlapping a gene. A single gene may thus have more than one 5′ read mapping region. (E) Examination of NanoPARE data from 2 replicates of WT and nrpd1−/− correctly identifies a documented miR159 cleavage site in MYB65 but identifies no difference in putative cleavage of the YUCCA10 transcript. YUCCA10 was chosen as an example because it shows increased mRNA abundance and reduced sRNA abundance in nrpd1−/−. (F) The relative distance metric shows no significant correlation between misregulated genes and sites losing sRNAs in nrpd1+/− and nrpd1−/−. Relative distance was calculated using bedtools. Black line indicates relative distance between sites losing sRNA (identified by DESeq2 by examination of read counts over 300-bp windows) and misregulated genes. Gray lines represent 5 replicates of equivalent number of random sites in the genome and misregulated genes. A uniform frequency of about 0.02 indicates no major correlation between the 2 datasets. 5,896, 1,720, and 790 sites lost sRNAs in nrpd1−/− [file pbio.3001602.s007.pdf]

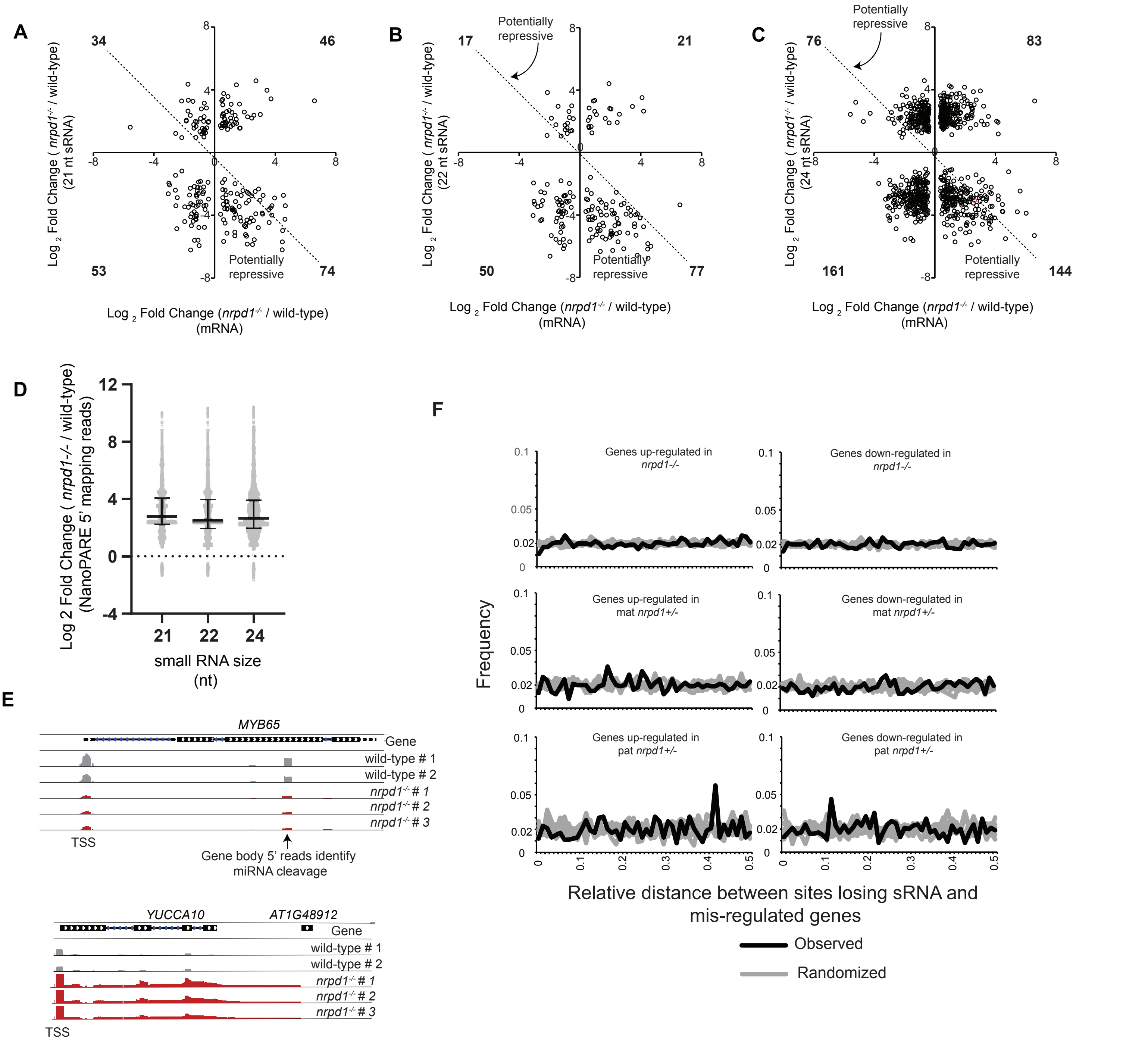

S7 Fig. Little relationship between Pol IV sRNAs and gene regulation.

(A-C) Comparisons of genes showing significant differences in 21, 22, 24 nt sRNA and mRNA abundance shows that only a subset of genes (lower right quadrant) may be repressed by Pol IV-dependent small RNAs in wild-type. Differences in small RNA abundance between wild-type and *nrpd1-/-* were calculated using DESeq2. Differences in mRNA was calculated using Cuffdiff. Numbers in bold in each quadrant indicate number of genes. **(D)** NanoPARE data maps 5' ends of transcripts and identifies transcriptional start sites (TSS) and cleavage sites within the gene body. Change in mRNA cleavage at genes that show increased mRNA abundance and decreased 21, 22 or 24 nt sRNA. Coverage of 5' end reads from NanoPARE sequencing was calculated for every nucleotide in the genome. Difference in 5' read coverage at each nucleotide was calculated for two replicates of WT endosperm (L*er* x Col) and three *nrpd1-/-* (L*er nrpd1-/-* x Col *nrpd1-/-*) replicates using DESeq2. Each point plotted on the dot plot represents one nucleotide with differential 5' reads overlapping a gene. A single gene may thus have more than one 5' read mapping region. **(E)** Examination of NanoPARE data from two replicates of wild-type and *nrpd1-/-* correctly identifies a documented miR159 cleavage site in *MYB65* but identfies no difference in putative cleavage of the *YUCCA10* transcript. *YUCCA10* was chosen as an example because it shows increased mRNA abundance and reduced small RNA abundance in *nrpd1-/-*. **(F)**The relative distance metric shows no significant correlation between mis-regulated genes and sites losing small RNAs in *nrpd1+/-* and *nrpd1-/-*. Relative distance was calculated using bedtools. Black line indicates relative distance between sites losing sRNA (identified by DESeq2 by examination of readcounts over 300 bp windows) and mis-regulated genes. Gray lines represent 5 replicates of equivalent number of random sites in the genome and mis-regulated genes. A uniform frequency of about 0.02 indicates no major correlation between the two data-sets. 5896,1720 and 790 sites lost sRNAs in *nrpd1-/-*, mat *nrpd1+/-* and pat *nrpd1+/-* respectively. The relative choppiness of the the distribution in pat *nrpd1+/-* is likely driven by the smaller number of sites being compared. Data represented in this figure can be seen in S6 Data.
