# Supplementary material for: RNA Pol IV induces antagonistic parent-of-origin effects on Arabidopsis endosperm
Source: PLoS Biol. 2022 Apr 7;20(4):e3001602. doi: 10.1371/journal.pbio.3001602 (PMC9017945; doi:10.1371/journal.pbio.3001602)
Supplement: S8 Fig — (A) Comparison of methylation in CG, CHG, and CHH contexts at individual cytosines within genes in WT. Cytosines in the first 2 columns on the left lie within misregulated genes whose sRNA abundances are up or down in nrpd1−/−. The control sets include cytosines within 5 randomly selected subset of genes that show no changes in mRNA abundance in nrpd1−/−. (B) Genes with fewer sRNAs in nrpd1−/− and misregulated expression tend to be longer. (C) Longer genes have more total 24-nt sRNAs in WT endosperm. (D) WT CHH methylation at sRNA-producing sites that are dependent on maternal or paternal Pol IV. Methylation is significantly higher at paternal Pol IV–dependent sites. (E) Effects of parental Pol IV loss on CHH methylation at regions with parental Pol IV–dependent sRNAs and greater than 10% CHH methylation in WT. Red, difference between mat nrpd1+/− and WT; blue, difference between pat nrpd1+/− and WT. sRNA-producing regions impacted in paternal nrpd1+/− have greater losses of CHH methylation. For D and E, CHH methylation was calculated for 300-bp sliding windows with a 200-bp overlap. CHH methylation windows overlapping windows losing sRNAs in nrpd1+/− endosperm were identified and merged using bedtools; maximum CHH methylation among merged windows was used for violin plot. *** represents a statistically significant difference as calculated by Wilcoxon test (p < 0.001). Boxplot in the violin plot shows median and interquartile range. (F) The relative distance metric shows no significant correlation between misregulated genes and sites with changes in CG and CHH DNA methylation in mat nrpd1+/−. Relative distance was calculated using bedtools. Black line indicates relative distance between misregulated genes and sites with differences in DNA methylation between WT and mat nrpd1+/− (identified by Bismark). Gray lines represent relative distance between 5 replicates of random sites in the genome and misregulated genes. A uniform frequency of about 0.02 indicates no major [file pbio.3001602.s008.pdf]

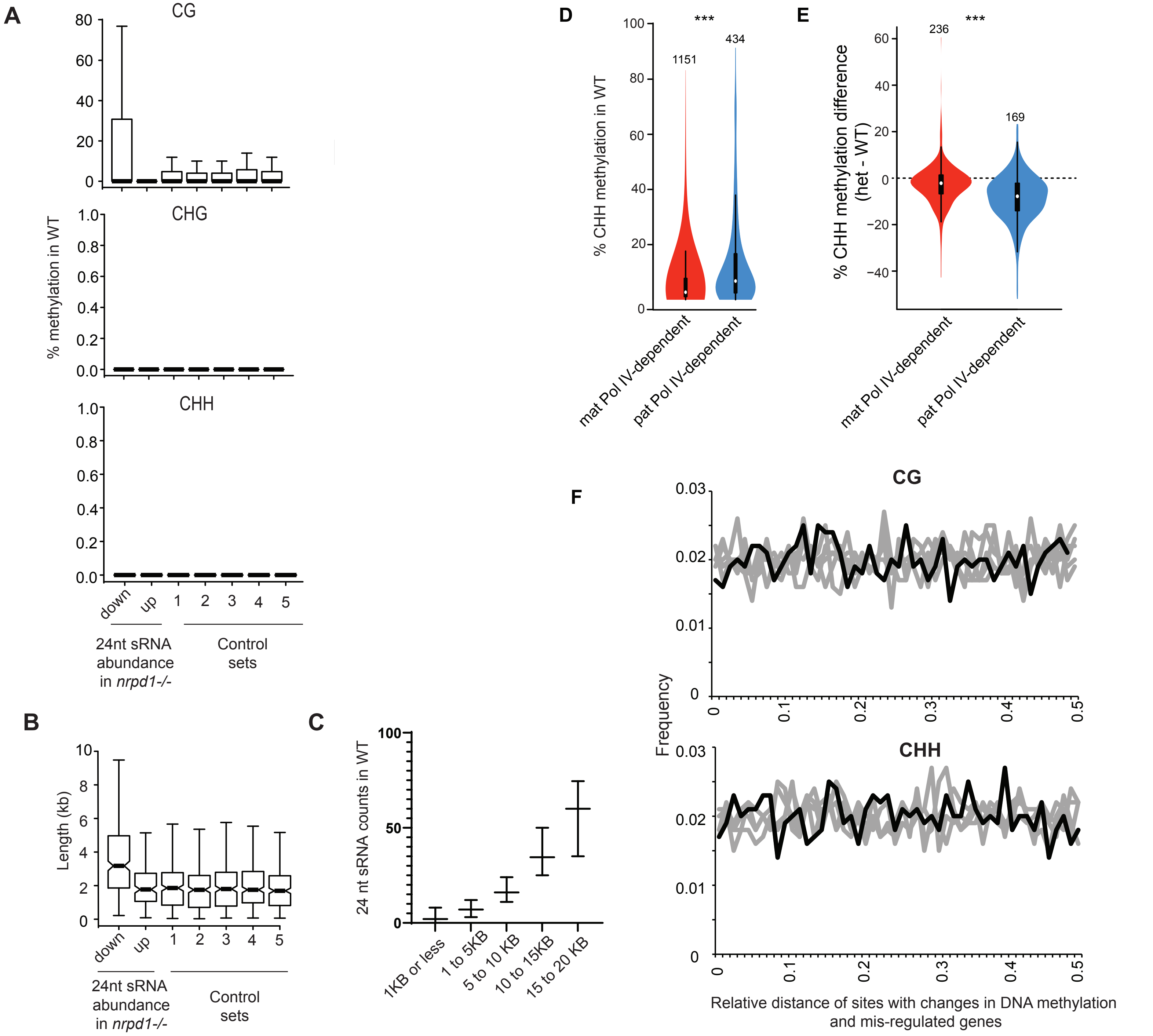

S8 Fig. No relationship between DNA methylation changes and genic mis-regulation in *nrpd1+/-*.

(A) Comparison of methylation in CG, CHG and CHH contexts at individual cytosines within genes in wild-type. Cytosines in the first two columns on the left are within mis-regulated genes whose sRNA abundances are up or down in *nrpd1-/-*. The control sets include cytosines within five randomly selected subsets of genes that show no changes in mRNA abundance in *nrpd1-/-*. **(B)** Genes with fewer sRNAs in *nrpd1-/-* and mis-regulated expression tend to be longer. **(C)** Longer genes have more total 24 nt sRNAs in wild-type endosperm. **(D)** Wild-type CHH methylation at sRNA-producing sites that are dependent on maternal or paternal Pol IV. Methylation is significantly higher at paternal Pol IV-dependent sites. **(E)** Effects of parental Pol IV loss on CHH methylation at regions with parental Pol IV-dependent sRNAs and greater than 10% CHH methylation in WT. Red, difference between mat *nrpd1+/-* and WT; blue, difference between pat *nrpd1+/-* and WT. Small RNA producing regions impacted in paternal *nrpd1+/-* have greater losses of CHH methylation. For D and E, CHH methylation was calculated for 300 bp sliding windows with a 200 bp overlap. CHH methylation windows overlapping windows losing small RNAs in *nrpd1+/-* endosperm were identified and merged using bedtools; maximum CHH methylation among merged windows was used for violin plot. *** represents a statistically significant difference as calculated by Wilcoxon test ($p<0.001$). Boxplot in the violin plot shows median and inter-quartile range. **(F)** The relative distance metric shows no significant correlation between mis-regulated genes and sites with changes in CG and CHH DNA methylation in mat *nrpd1+/-*. Relative distance was calculated using bedtools. Black line indicates relative distance between mis-regulated genes and sites with differences in DNA methylation between wild-type and mat *nrpd1+/-* (identified by Bismark). Gray lines represent relative distance betwen 5 replicates of random sites in the genome and mis-regulated genes. A uniform frequency of about 0.02 indicates no major correlation between the two datasets. Data represented in this figure can be found in S7 Data.
