# Supplementary material for: RNA Pol IV induces antagonistic parent-of-origin effects on Arabidopsis endosperm
Source: PLoS Biol. 2022 Apr 7;20(4):e3001602. doi: 10.1371/journal.pbio.3001602 (PMC9017945; doi:10.1371/journal.pbio.3001602)
Supplement: S9 Fig — (A) Genes were examined to identify those whose expression differences were driven by allele-specific effects. Genes with at least a 2-fold, statistically significant difference in expression between the indicated heterozygote and WT and at least 10 allele-specific reads in both genotypes were included. The shift in allelic expression was evaluated by subtracting the % maternal allele transcripts in WT from the heterozygote. Genes within Col-0 introgressions that remain in Ler nrpd1−/− plants were excluded from all analyses. (B) Examples of genes showing allele-specific impacts upon loss of maternal Pol IV. FPKM and fold change in (A) and (B) are from Cuffdiff output. Data represented here can be found in S8 Data. FPKM, fragments per kilobase of exon per million; Pol IV, polymerase IV; WT, wild type. (PDF) [file pbio.3001602.s009.pdf]

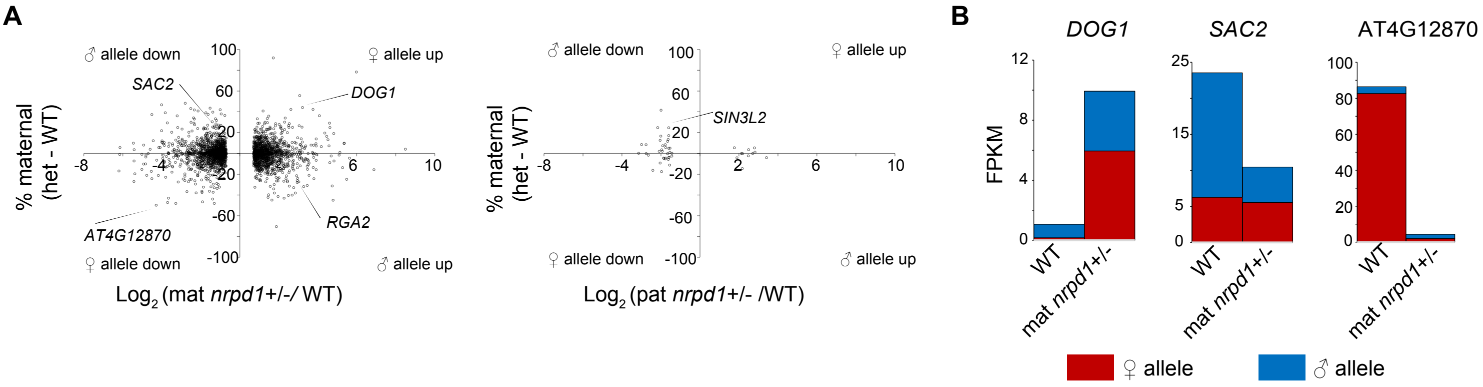

S9 Fig. Impact of parental *NRPD1* on maternal and paternal allele contributions to total gene expression.
(A) Genes were examined to identify those whose expression differences were driven by allele-specific effects. Genes with at least a two-fold, statistically signficant difference in expression between the indicated heterozygote and WT and at least ten allele-specific reads in both genotypes were included. The shift in allelic expression was evaluated by subtracting the % maternal-allele transcripts in WT from the heterozygote. Genes within Col-0 introgressions that remain in L*er nrpd1*-/- plants were excluded from all analyses. **(B)** Examples of genes showing allele-specific impacts upon loss of of maternal Pol IV. FPKM and fold-change in (A) and (B) are from Cuffdiff output. Data represented here can be found in S8 Data.
