# Supplementary material for: RNA Pol IV induces antagonistic parent-of-origin effects on Arabidopsis endosperm
Source: PLoS Biol. 2022 Apr 7;20(4):e3001602. doi: 10.1371/journal.pbio.3001602 (PMC9017945; doi:10.1371/journal.pbio.3001602)
Supplement: Attachment — Submitted filename: 119879_1_rebuttal_2248979_r46c1g.pdf [file pbio.3001602.s030.pdf]

Response to reviewers:

Reviewer #1

I believe that this is a very sound and authoritative study. The analysis of all data seems appropriate and robust, and many connections between the data (and subsets of data) and their possible interpretations have been considered. In fact, in the massive Results section, some interpretations are supported by cited references (this is not meant as a critique). However, I wonder about the length of the Results section, and the balance between it and the relatively short Discussion section. It is difficult for me to nail down any part of Results that might be shortened, as I could not find clear redundancies. I also think that the level of speculation is absolutely warranted, and I did not find excessive claims being made to this or that end. Rather, I suggest to broaden the perspective somewhat (in their Discussion; see below under Significance), which might allow people with a less mechanistic perspective to grasp the potential relevance of this work for non-model plant systems studied mostly by evolutionary geneticists.

Response: We thank the reviewer for their kind remarks. We have spent a very large amount of time trying to streamline the results section and we are not sure if it would be possible to shorten it any further without removing critical details.

We appreciate the reviewer's comment to add more detail to the discussion to make it more appealing to evolutionary geneticists and we have added the following lines to the discussion section: "The WISO or "weak inbreeder/strong outcrosser" model (Brandvain & Haig, 2005) emerges from the dynamics of parental conflict and parent-of-origin effects. Under this model, a parent from populations with higher levels of outcrossing is exposed to higher levels of conflict and can thus dominate the programming of maternal resource allocation in a cross with an individual from a population with lower levels of outcrossing. Such a phenomenon has been observed in numerous clades including *Dalechampia*, *Arabidopsis*, *Capsella* and *Leavenworthia* (Brandvain & Haig, 2018; İltaş et al., 2021; Lafon-Placette et al., 2018; Raunsgard et al., 2018). Intriguingly, loss of function phenotypes in the RdDM pathway are more severe in recently outcrossing species than in *A.thaliana* (Grover et al., 2018; Wang et al., 2020) and suggests that RNA Pol IV functions are more elaborate and important in these species. This raises the possibility that the role for RNA Pol IV and RdDM in parental conflict that we describe in *A.thaliana* here is likely heightened in and mediates the elevated level of parental conflict in species that are currently or have been recently outcrossing."

One aspect that might warrant more scrutiny is the mapping of sRNA reads to the reference genome. I found the short section of this (M&M section, page 20, lines 23-25) to be too brief. It is not clear to me which of ShortStack's v3 weighting scheme the authors used, which is relevant for multi-mapping reads (see NR Johnson et al. 2016, G3). In addition, it is not mentioned whether zero mismatches were allowed. Perhaps this is described in more detail in Erdmann et al. (2017), but even if so, it deserves to be clarified here.

Response: Small RNA reads were aligned after allowing two mismatches. This was indicated in the bowtie command ('*bowtie -v 2'* where v 2 indicates two mis-matches). We have added text to expand on the meaning of the commands.

We have also expanded the commands used for ShortStack. We used the "Placement guided by uniquely mapping reads (-u)" option to divide the multi-mapping reads.

The manuscript is well-written and concise, despite the length of the Results section. The verbal clarity and absence of typos or grammatical issues is superb. I did find some of the Figures to be somewhat "un-intuitive", in the sense that it takes acute concentration for an outsider (of sorts) to gather and interpret the underlying data. This is probably due to the many cross-comparisons of differences between two genotypes on one axis and those of a different pair of genotypes on the other axis. I am not sure how this issue can be ameliorated (nor whether this is really necessary); however, from a technical point of view, all Figures and Suppl. Figures are really well-done.

Response: We thank the reviewer for their kind remarks. We have strived to make the figures easier to understand but we are aware that the figures do require a lot of concentration. We haven't found an easy way to fix this. We thank the reviewer for patiently going through the figures.

The list of references seems adequate in terms of citing relevant (both older and very recent) publications. However, almost all cited papers concern Arabidopsis or other model species; I suggest to consider adding a few relevant studies on non-Brassicaceae (whether considered model taxa or not), in conjunction with my suggestion (in Significance) to potentially broaden the scope by searching for natural phenomena that also involve parent-of-origin effects on endosperm/seed development. Curiously, many of the references are "incomplete" in the sense of stopping with the journal's name, then stating the doi, i.e. they lack volume numbers and page/article numbers. This should be harmonized throughout.

Response: We have added references to non-Brassicaceae species and have also fixed the references.

Reviewer #2:

This manuscript provides evidence that a loss of either the maternal or paternal copy of NRPD1 have different, and sometime opposite, effects on the accumulation of small RNAs and on expression of a subset of genes, with a loss of the maternal copy having more substantial effects. The manuscript is well written, and the conclusions, as far as they go, are justified by the data, which are effectively presented. The overall effect is subtle but informative and according to the authors support a parental conflict model for imprinting. The experiments failed to find a smoking gun in the form of a mechanism to explain how or why the maternal and paternal alleles have different effects and the explanation for a lack of clear phenotypic differences was reasonable, but untested. I would have like to see it tested by looking in a plant species that is outcrossing and highly polymorphic. However, I do appreciate that the observation that the male and female alleles can have distinct effect when mutant is an important clue. My specific comments below may reflect confusion on my part, rather than real issues. If that is that I hope that confusion can aid in clarifying what are in places quite subtle points.

Response: We thank the reviewer for their comments. We agree that it would be potentially informative to do similar experiments in an outcrossing species but that this is beyond the scope of this manuscript. Additionally, loss of *NRPD1* or other components of the RdDM pathway has dramatic effects on gametogenesis in some examined outcrossing species(Grover et al., 2018;

Wang et al., 2020), which could prevent the detection of subtle parent-of-origin effects on seed development.

Page 6, last paragraph: "Because the endosperm is triploid, in these comparisons there are 3 (wild-type), 2 (pat nrpd1+/-), 1 (mat nrpd1+/-) and 0 (nrpd1-/-) functional NRPD1 alleles in the endosperm. However, NRPD1 is a paternally expressed imprinted gene in wild-type Ler x Col endosperm and the single paternal allele contributes 62% of the NRPD1 transcript whereas 38% comes from the two maternal alleles (Pignatta et al., 2014). Consistent with paternal allele bias in NPRD1 expression, mRNA-Seq data shows that NRPD1 is expressed at 42% of wild-type levels in pat nrpd1+/- and at 91% of wild-type levels in mat nrpd1+/- (Supplementary Table 6)".

I would think this would really complicate the analysis. Should all of the dosage values include NPRD1 imprinting values? That is to say, expressed in terms of expression values? This is also a bit confusing. The maternal copies together express 38% of the transcript, so why isn't the mat nrpd1 at 68%, rather than 91%? In any event, given this imprinting and differences in dosage of the male and female it appears that two variables, parental origin and expression levels are being compared. Since 91% is awfully close to 100%, are the mat pat comparisons really just comparing low with nearly normal expression of NRPD1? And actually, given that, the outsized effect of the mat nrpd1 +/- is even more striking.

Response: We included the details of dosage rather than imprinting values because the potential for buffering of expression upon loss of one allele could not be discounted. Indeed, we do find that the endosperm transcriptome buffers against the loss of the maternal or paternal alleles (Supplementary Table 6). The reviewer is correct in pointing out that the outsized effect of mat *nrpd1+/-* on gene expression is even more striking, and strongly supports our view that these effects are parental rather than endospermic.

To reduce confusion in this section, we removed the details about 38% maternal allele transcripts obtained from our previous study, and instead report only the observed values from this study (which are also consistent with the previously reported paternally-biased expression of *NRPD1* in endosperm).

Page 4, Line 16. I'm afraid it's still a bit difficult to understand what was being compared what in this section. Please clarify.

Response: The authors in this previously published study compared sRNAs obtained from wild-type whole seeds (which consists of three different tissues, including endosperm) with mutant endosperm. We are pointing out that the difference in tissue composition makes the effect of *nrpd1* mutation hard to disentangle from the tissue differences between the two genotypes.

Page 5, Line 5. I'm sure this is fine, but it's not entirely clear what is from the previously published paper and what is reanalysis here. All the crosses and measurements were made then, but not organized in this way?

Response: This data was indeed previously published. In that analysis, we had pooled results from different crosses and calculated significance between genotypes using chi-square tests. During a later study (Satyaki and Gehring, 2019), we realized that we were losing information by ignoring the seed abortion values per cross. So, a reanalysis of that data on a cross by cross basis allowed us to find strong evidence for maternal and paternal effects.

Page 6, Line 26. This is an excellent dosage series, but it's complicated by imprinting. So it's not 3, 2, 1, 0 effective copies. If we set the paternal copy at ~1 and each maternal at ~0.1, then it's 1.2 (wild type), 0.20 (pat nrpd1+/-), 1 (mat nrpd1+/-), and 0 (nrpd1-/-).

Response: At the genomic DNA level, its 3, 2,1 and 0 doses. The reviewer's comment on the transcriptional dose is not clear to us. Based on measured gene expression levels, relative wild-type *NRPD1* transcriptional dose =1, pat *nrpd1+/-* is 0.42, and mat *nrpd1+/-* is 0.91.

Page 6, line 31. Is the main thing we are comparing the difference between expression at 42% verses 91% of wild type?

Response: We are using the small RNA-seq data alongside the mRNA-seq data to argue that loss of mat and pat nrpd1+/- have no impact on overall Pol IV activity *in endosperm* (as measured by small RNA production). A *nrpd1* heterozygous endosperm has almost the same small RNA profile as a wild-type endosperm. Thus any effects seen in the endosperm, including the effects on mRNA expression described later in the manuscript, are likely parental rather than zygotic endospermic effects.

Page 7, line 11. So, the overall effect in either direction on smRNA gene targets was really quite small, and I'm guessing the effect on gene expression was even smaller.

Response: The effects of loss of maternal or paternal Pol IV on sRNAs was indeed small (Fig. 1/Fig. S3). Effect of loss of maternal Pol IV on gene expression was substantially large and distinct from the relatively small impacts observed upon loss of paternal Pol IV (Fig. 3) This observation supports the view that Pol IV mediates parent-of-origin effects on gene expression.

Page 7, line 17. I take it that it is this difference, rather than the overall numbers that is of interest.

Response: Correct. The lack of a relationship between sRNAs impacted upon loss of mat and pat *nrpd1* is additionally suggestive of parent-of-origin effects

Page 9, line 2. Really interesting, since one might expect that these are methylated loci that would be expected to be fed into any existing embryo maintenance methylation pathway. Surprising that they are maintained independently.

Response: It is indeed surprising that Pol IV activity in parents can have different impacts on sRNAs in the endosperm. It should be noted though, that as described in Erdmann et al 2017 and in this paper later on, many endosperm sRNA loci are in fact not associated with endosperm DNA methylation. In addition, sRNA loci that are dependent on paternal Pol IV activity are more likely to be associated with DNA methylation than are sRNA loci associated with maternal Pol IV activity. These points have been described in Figure S8.

Page 9, line 22. Proportion of total imprinted genes? Did the mutant obviate/enhance the imprinting?

Response: We have modified the manuscript to describe effects on imprinted genes: “ The expression of imprinted genes is known to be regulated epigenetically in endosperm. In mat *nrpd1+/-* imprinted genes were more likely to be mis-regulated than expected by chance (hypergeometric test $p<10^{-15}$) – 15 out of 43 paternally expressed and 45 out of 128 maternally expressed imprinted genes were mis-regulated in mat *nrpd1+/-* while two maternally expressed imprinted genes but no paternally expressed imprinted genes were mis-regulated in pat *nrpd1+/-* (Table S6).” We have also added a new supplementary figure (Fig. S6) that describes the impacts of *NRPD1* loss of imprinted gene expression.

Page 9, line 27. How could 2) occur in the homozygous mutant?

Response: Loss of *NRPD1* may impact gene expression in both parents. When the *nrpd1-/-* mutant endosperm is investigated, we are also examining the consequences of the inheritance of these disrupted gene expression states. We refer to this as epistatic interactions of mat and pat *nrpd1*.

Page 10, line 9. Interesting!

Response: We strongly agree!

Page 10, line 11. Is this 2.7 versus 2.18 significant because it's statistically significant, or because it's conceptually significant?

Response: We are pointing out that the 2.7-fold value is quite similar to the predicted value of 2.18-fold, which is arrived at by simply summing the effects of mat *nrpd1* and pat *nrpd1*. This is a conceptually significant point.

Are the examples in 3D representative, or the most convincing examples? And a big difference in ROS1 is of some concern, since that may well be expected to affect imprinting indirectly. I know I'm being picky here, but the pattern is so intriguing I'd be worried about confirmation bias.

Response: The examples in 3D are representative for those genes with significant changes in expression in both mat and pat *nrpd1*, and other genes also behave similarly. The antagonistic effect described for 3D can also be observed as a much broader trend affecting hundreds of genes to varying extents in Fig 3C and 3E-H. The concern about *ROS1* is not clear to us but we agree that an effect of *ROS1* may be one way that *NRPD1* controls gene expression.

Page 10, line 18. Ok, but 0.123 is a pretty subtle negative correlation. Although I do appreciate that it clearly is not a positive correlation. If I'm understanding correctly, this was the "aha" moment, because it's exactly what one might expect of NRPD1 from the mother and father or working at cross purposes. But the numbers are getting awfully small here.

Response: It is unclear how to calibrate our expectations of effect sizes considering that our study is the first (to our knowledge) to make such a measurement involving gene expression in parental conflict. A review of the few empirical examples of parental conflict’s impact on seeds shows that parental conflict may drive small changes in seed size (Brandvain and Haig, 2018).

The evolution of quantitative traits maybe driven by selection for large effects at a small number of loci and/or by selection of small effects at a large number of loci. In a similar vein, parental conflict can impact seed phenotypes either via large effects at a few loci or via small effects at a large number of loci. Our analysis described in Fig 3D-H can fit either possibility.

Large effects can be found at a few loci such as *SUC2* and *PICC* (Fig. 3D). Smaller antagonistic effects can be found at hundreds of loci as shown in Figure 5A. The negative correlation described in this figure can be observed even upon dropping the genes that show a statistically significant differential expression in both mat and pat *nrpd1+/-* (slope after dropping genes significantly mis-regulated in both mat and pat *nrpd1+/-* is -0.126). In summary, a correlation of -0.123 strongly supports the existence of a widespread antagonistic regulatory effect.

Page 10, line 29. The point simply being that that other phenomenon is also significant even if the differences are that large?

Response: We are pointing out that the magnitude of the effects we see are similar to that observed for phenomenon such as dosage compensation.

Page 12. So, there is no effect on cleavage and no obvious effect on flanking siRNA clusters. The suspense is building...

…

Page 12, line 24. And not in potential regulatory regions? CNSs?

Response: We did not identify a significant enrichment for differentially methylated regions in regulatory regions. We used the relative distance function in bedtools (https://bedtools.readthedocs.io/en/latest/content/tools/reldist.html) to calculate the relationship between the genomic location of DMRs and genomic location of a differentially expressed gene. This analysis was chosen as it does not make *a priori* assumptions about the size of the regulatory region of a gene. A broad association between DMRs and differentially expressed genes would be indicated by a frequency far greater than 0.02. We show the results of this analysis in Fig. S8F; we find no evidence for significant enrichment of DMRs in the regulatory regions of differentially expressed genes.

Page 12, line 28. I guess it depend on whether or not the changes are in regulatory sequences no immediately apparent as part of the gene, doesn't it?

Response: We examined DNA methylation over genes here because in endosperm, unlike in other tissues, many small RNAs are genic. Moreover, DNA methylation within the gene may control transcript abundance (Eimer et al., 2018; Klosinska et al., 2016). We have also examined regulatory regions adjacent to genes in Fig S8F and found no effect.

Line 13, line 2. Not sure it's that important, but couldn't you chop all of these genes in half and see if they are no longer significant collectively?

Response: We do not think that this will provide a useful insight.

Page 14, line 15. I'm afraid I'm getting confused here with the terms cis and trans here. Just to be clear, cis means a direct effect of small RNAs that are dependent on NRPD1 on a gene and trans means anything else? But in this context, it's not clear that is what is meant. Do you mean that gene expression is determined and preset in the gametophyte? What are the levels of expression of NRPD1 in the two gametophytes?

Response: The reviewer's interpretation of cis and trans is correct. However, the cis imprints may be preset in gametophytes or in the sporophytic tissues that surround or give rise to the

gametophyte. Pol IV is known to be active either in gametophyte or in related sporophytic tissues in both the mother and the father(Kirkbride et al., 2019; Long et al., 2021; Olmedo-Monfil et al., 2010).

Page 14, line 19. Prior to fertilization?

Response: Yes, that is the idea. As described in the manuscript, Pol IV activity in either the parental sporophyte or gametophyte prior to fertilization could impact gene expression in the endosperm after fertilization.

Page 14, line 27. Do you mean driven by, or just associated with?
Response: In response to the comment, we have replaced the phrase "driven by" with "due to" for increased clarity. In wild-type, *DOG1* is predominantly expressed from the paternal allele. In mat *nrpd1+/-*, the paternal allele is somewhat upregulated but the maternal allele, which is almost silent in wild-type, is highly expressed in mat *nrpd1+/-*.

Page 15, line 26. And this is really the issue. The primary conclusion, backed up by the lack (I'm assuming) of phenotypic differences between mat NRPD1 -/+ and pat NRPD1 +/- suggests that the observed differences in expression are not particularly important, unless the exceptional cases are informative.

Response: We are not sure whether the reviewer means "issue" in a negative, neutral, or positive light. Seed phenotypes are often subtle and we have not examined phenotypic differences in sufficient detail to comment.

Page 15, line 12. Yes, but I'm not at all clear what the mechanism for this is.

Response: We have tested and falsified multiple hypotheses to explain how Pol IV can regulate gene expression in endosperm. Considering the complex genetics and the difficulty of isolating endosperm, we have concluded that this is a matter for a future study. The point of this study is the discovery of Pol IV's parental effects.

Page 15, line 23. Since this is a very small subset of genes, are these genes that you might expect to play a role in parental conflict?

Response: The functions of most genes in endosperm remain unknown. However, some have a likely role in conflict. *SUC2* is antagonistically regulated by parental Pol IV (Fig. 3D). SUC2 transports sucrose, the key form of carbon imported into seeds from the mother (Sauer & Stolz, 1994).

Page 15, line 33. Indeed, these could be the informative exceptions.

Response: We believe the reviewer means that the identify of strongly antagonistically regulated genes may be informative in terms of thinking about these results in the context of parental genetic conflict, which we agree with.

Page 15, line 29. Hardly surprising, given that the paternal copy of NRPD1 is expressed at a higher level than the maternal copies, is it?
Response: It is actually somewhat surprising since we show in Fig. 2 that the sRNA production in mat and pat *nrpd1+/-* are comparable to that of wild-type. The higher contribution of *NRPD1* from the paternal copy does not really explain the methylation differences

Page 16, line 1. So this is what you mean by in cis. Presetting?

Response: The reviewer's previous interpretation of *cis* (acting directly at a target gene) is correct. However, the *cis* imprints may be preset in gametophyte or in the sporophytic tissues that surround or give rise to the gametophyte. Pol IV is known to be active in gametophytes and in related sporophytic tissues in both the mother and the father.

These are intriguing results that would benefit from a test of the hypothesis by comparing these result with those obtained in an outcrossing plant species.

Response: We agree that it would interesting and informative to perform similar experiments in an outcrossing species. However, loss of *NRPD1* or other components of the the RdDM pathway have dramatic effects on gametogenesis in outcrossing species (Grover et al., 2018; Wang et al., 2020), preventing the detection of subtle parent-of-origin effects on seed development. Additionally, this would be a separate study.

Reviewer #3

We thank the reviewer for their comments.

- Expression of NRPD1 was 42% of WT in paternal nrpd1 and 91% of WT in maternal nrpd1, yet throughout the paper the effect of maternal nrpd1 was far stronger than paternal nrpd1. The authors may also want to confirm that protein levels follow the same pattern, in case protein degradation or post-transcriptional regulation may play a role.

Response: We show in Fig. 2 that sRNA production in mat and pat *nrpd1+/-* are similar to wild-type endosperm. This strongly suggests that *NRPD1* protein is produced at functionally equivalent levels in wild-type, mat and pat *nrpd1+/-.* The finding that mat *nrpd1+/-* has a stronger effect on gene expression and small RNAs, despite having higher levels of *NRPD1* transcript in endosperm, is consistent with our conclusion that the effects we are observing in heterozygous endosperm are due to *NRPD1* action before fertilization.

- P. 9 line 1 - this only seems to be true for maternal ISRs, not paternal ISRs; this claim should be narrowed.
Response: Accordingly, we have modified the text here to : "In summary, these results indicate that most maternally and some paternally imprinted sRNA loci in endosperm are dependent on Pol IV activity in the parents and are not established *de novo* post-fertilization."

A small number of sRNA loci become highly depleted in maternal nrpd1 but not paternal nrpd1 (Fig. 1D, F, Fig. 2C) - are these siren loci?

Response: This is an interesting question. Siren loci have not been defined in Arabidopsis but are described as loci with high levels of sRNAs in ovules, seed coat, endosperm and embryo (Grover et al., 2020). Loci losing sRNAs in maternal *nrpd1+/-* include a large number of maternally expressed

imprinted sRNAs (mat ISRs). We do not know if mat ISR loci are expressed in the ovule. In Erdmann et al (2017), we excluded loci that were also expressed in the seed coat from mat ISRs. Thus, these loci meet only some of the conditions for being defined as siren loci.

Fig. 2 suggests that many of the downregulated sRNA regions in maternal nrpd1 are maternally biased to begin with. Related, are genic sRNAs more likely to be affected by maternal or paternal nrpd1 than non-genic or TE sRNAs?

Response: As described in Fig. 1B and S3, loss of maternal *NRPD1* has more impacts on the sRNA landscape. As a percentage of total loci, genes are more likely to be affected than TEs.

- For the sRNA loci shown in Fig. 2C, how is % maternal affected in maternal vs. paternal nrpd1? These ISRs are normally maternal or paternal biased, does this change in maternal or paternal nrpd1?

Response: We assess the allelic bias of ISRs only when they have at least ten reads in the genotypes being compared. In mat *nrpd1*+/-, most mat ISRs lose almost all their reads (Fig. 2) and we can assess allelic bias only at 107/366 mat ISRs. As seen in the Rev. comment. Fig1, these 107 lose their maternal bias. In pat *nrpd1+/-,* loci with maternally biased sRNAs show somewhat increased expression (Fig 2E) but do not show an appreciable change in maternal bias (Figure Review 1). All paternal ISRs do not show any dramatic impacts on allelic bias in mat or pat *nrpd1+/-.* We have not added this additional datapoint to our paper because we were worried that the paper was becoming too dense – a concern also voiced by reviewer 1. However, we can add this to the manuscript if the reviewer prefers.

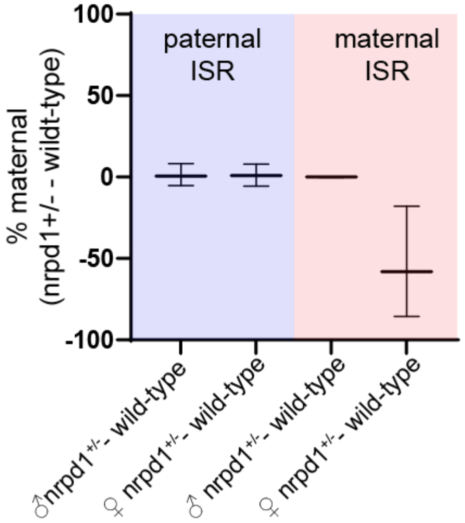

Reviewer comment Fig 1: Change in allelic origin of sRNAs in imprinted sRNA regions in *nrpd1+/-*. Graph shows shift in the allelic origin of sRNAs in ISRs in heterozygotes (median with interquartile range). To be included here, an ISR needed to have at least ten allele-specific reads in heterozygote and wild-type.

- Might have missed this, but I didn't see the gene ontology results (p9 line 16) shown anywhere? Would like to see significance values, fold enrichments, etc. In particular, the group of paternal nrpd1 up-regulated genes seems too small to have much confidence for GO enrichment analysis.

Response: We have added a Supplementary Table 7 with outputs of GO analyses.

- I would suggest expanding the analysis in Fig. 3D-H to explore whether the additive model is more predictive of nrpd1-/- expression levels than other potential models (epistatic, etc.) in general at all genes, or only at the subsets of genes shown, independently of whether the effects are large enough to pass the arbitrary significance cutoffs used in E-H. Identifying specifically which genes do and don't follow this additive pattern could help dissect mechanism. For example, genes following this pattern might share a TF binding site for a TF that is regulated by Pol IV.

Response: While we are interested, we currently cannot explore other models such as epistasis as this would require knock-down of NRPD1 in the endosperm and we plan to do this as part of a future study.

P. 13 line 26 - how do changes in CG methylation in maternal or paternal nrpd1 compare to changes in dme or ros1? Do either set of DMRs significantly overlap dme or ros1 DMRs? Could some of these be explained by changes in ROS1 expression, since ROS1 is a Pol IV target?

Response: Yes. It's entirely possible that a subset of observed gene expression changes are linked to changes in *ROS1* expression. However, there are no comparable methylation data for *ROS1* in the endosperm. A potential role for ROS1 has been discussed on Page 11, line 4. Comparison with DMRs in the *dme* endosperm is difficult. *dme* mutant endosperm has low non-CG methylation (Ibarra et al., 2012). We have unpublished data showing that the expression of genes involved in RNA-directed DNA methylation (RdDM) is reduced in the *dme* endosperm. It is therefore difficult to understand if and how DME-mediated demethylation may impact RdDM.

- P. 10 line 3 - is the overlap of 36 out of 51 genes unlikely to occur by chance?
Response: A hypergeometric test indicates that this is indeed significant. We have added it to text on Page 9, line 34.

- In sRNA and mRNA-seq libraries, what was the overall maternal/paternal ratio in each library? Did loss of Pol IV affect this?

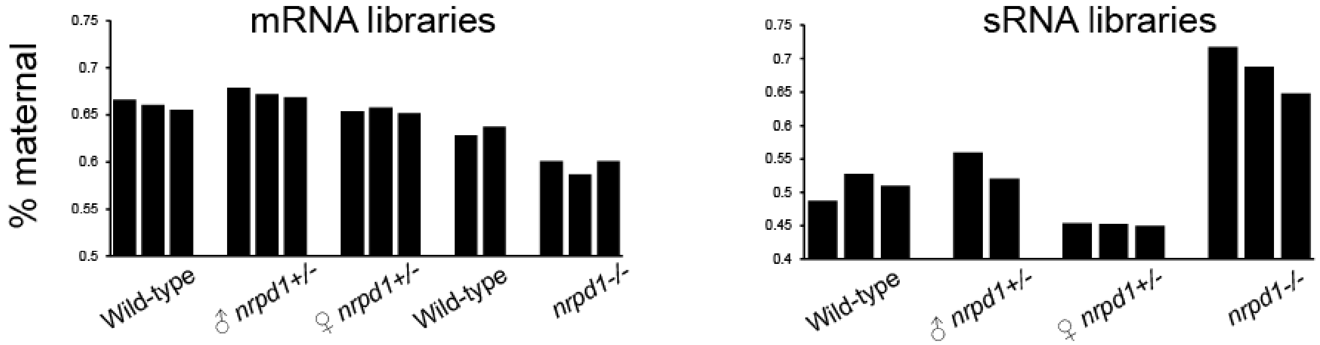

The graphs above show the maternally derived fraction of mRNA and sRNA libraries for different genotypes. Please note that the Ler *nrpd1* mutant was generated by backcrossing Col-0 *nrpd1+/-* into L*er*. Some Col-0 regions remain in this background and are called "hold-outs". Reads mapping to these hold-outs have been excluded while calculating the maternal fraction of each library described in the graph above. We cannot confidently judge if the overall maternal fraction of the mRNA transcriptome is affected by loss of *NRPD1* as we likely need more replicates. However, we find that loss of all NRPD1-dependent sRNAs (as in the *nrpd1* null mutant) leaves behind sRNAs that roughly reflect the genomic 2:1 ratio.

- P. 9 line 22 - how many paternally and maternally expressed imprinted genes were considered? Were imprinted genes statistically more likely to be misregulated in mat nrpd1?

Response: We considered 128 maternally and 43 paternally expressed genes that had been previously been identified as imprinted in Col x Ler crosses (Pignatta et al 2014). We have modified the manuscript to describe effects on imprinted genes: " The expression of imprinted genes is known to be regulated epigenetically in endosperm. In mat *nrpd1+/-* imprinted genes were more likely to be mis-regulated than expected by chance (hypergeometric test $p<10^{-15}$) – 15 out of 43 paternally expressed and 45 out of 128 maternally expressed imprinted genes were mis-regulated in mat *nrpd1+/-* while two maternally expressed imprinted genes but no paternally expressed imprinted genes were mis-regulated in pat *nrpd1+/-* (Table S6). " We have also added a supplementary figure (Figure S6) that focuses on genic mRNA imprinting in *NRPD1* heterozygotes and homozygous mutants.

References cited in the response

Brandvain, Y., & Haig, D. (2005). Divergent Mating Systems and Parental Conflict as a Barrier to Hybridization in Flowering Plants. *The American Naturalist*, *166*(3), 330–338. https://doi.org/10.1086/432036

Brandvain, Y., & Haig, D. (2018). Outbreeders pull harder in a parental tug-of-war. *Proceedings of the National Academy of Sciences*, *115*(45), 11354–11356. https://doi.org/10.1073/pnas.1816187115

Eimer, H., Sureshkumar, S., Singh Yadav, A., Kraupner-Taylor, C., Bandaranayake, C., Seleznev, A., Thomason, T., Fletcher, S. J., Gordon, S. F., Carroll, B. J., & Balasubramanian, S. (2018). RNA-Dependent Epigenetic Silencing Directs Transcriptional Downregulation Caused by Intronic Repeat Expansions. *Cell*. https://doi.org/10.1016/j.cell.2018.06.044

Grover, J. W., Burgess, D., Kendall, T., Baten, A., Pokhrel, S., King, G. J., Meyers, B. C., Freeling, M., & Mosher, R. A. (2020). Abundant expression of maternal siRNAs is a conserved feature of seed development. *Proceedings of the National Academy of Sciences of the United States of America*, *117*(26), 15305–15315. https://doi.org/10.1073/pnas.2001332117

Grover, J. W., Kendall, T., Baten, A., Burgess, D., Freeling, M., King, G. J., & Mosher, R. A. (2018). Maternal components of <scp>RNA</scp> -directed <scp>DNA</scp> methylation are required for seed development in *Brassica rapa*. *The Plant Journal*, *94*(4), 575–582. https://doi.org/10.1111/tpj.13910

Ibarra, C. A., Feng, X., Schoft, V. K., Hsieh, T.-F., Uzawa, R., Rodrigues, J. A., Zemach, A., Chumak, N., Machlicova, A., Nishimura, T., Rojas, D., Fischer, R. L., Tamaru, H., & Zilberman, D. (2012). Active DNA Demethylation in Plant Companion Cells Reinforces Transposon Methylation in Gametes. *Science*, *337*(6100), 1360–1364. https://doi.org/10.1126/science.1224839

İltaş, Ö., Svitok, M., Cornille, A., Schmickl, R., & Lafon Placette, C. (2021). Early evolution of reproductive isolation: A case of weak inbreeder/strong outbreeder leads to an intraspecific hybridization barrier in Arabidopsis lyrata. *Evolution*, *75*(6), 1466–1476. https://doi.org/10.1111/evo.14240

Kirkbride, R. C., Lu, J., Zhang, C., Mosher, R. A., Baulcombe, D. C., & Chen, Z. J. (2019). Maternal small RNAs mediate spatial-temporal regulation of gene expression, imprinting, and seed development in Arabidopsis. *Proceedings of the National Academy of Sciences*, *116*(7), 2761–2766. https://doi.org/10.1073/pnas.1807621116

Klosinska, M., Picard, C. L., & Gehring, M. (2016). Conserved imprinting associated with unique epigenetic signatures in the Arabidopsis genus. *Nature Plants*, *2*, 16145. https://doi.org/10.1038/nplants.2016.145

Lafon-Placette, C., Hatorangan, M. R., Steige, K. A., Cornille, A., Lascoux, M., Slotte, T., & Köhler, C. (2018). Paternally expressed imprinted genes associate with hybridization barriers in Capsella. *Nature Plants*, *4*(6), 352–357. https://doi.org/10.1038/s41477-018-0161-6

Long, J., Walker, J., She, W., Aldridge, B., Gao, H., Deans, S., Vickers, M., & Feng, X. (2021). Nurse cell-–derived small RNAs define paternal epigenetic inheritance in Arabidopsis. *Science*, *373*(6550). https://doi.org/10.1126/science.abh0556

Olmedo-Monfil, V., Durán-Figueroa, N., Arteaga-Vázquez, M., Demesa-Arévalo, E., Autran, D., Grimanelli, D., Slotkin, R. K., Martienssen, R. A., & Vielle-Calzada, J.-P. (2010). Control of female gamete formation by a small RNA pathway in Arabidopsis. *Nature*, *464*(7288), 628–632. https://doi.org/10.1038/nature08828

Raunsgard, A., Opedal, Ø. H., Ekrem, R. K., Wright, J., Bolstad, G. H., Armbruster, W. S., & Pélabon, C. (2018). Intersexual conflict over seed size is stronger in more outcrossed populations of a mixed-mating plant. *Proceedings of the National Academy of Sciences*, *115*(45), 11561–11566. https://doi.org/10.1073/pnas.1810979115

Sauer, N., & Stolz, J. (1994). SUC1 and SUC2: two sucrose transporters from Arabidopsis thaliana; expression and characterization in baker's yeast and identification of the histidine-tagged protein. *The Plant Journal*, *6*(1), 67–77. https://doi.org/10.1046/j.1365-313X.1994.6010067.x

Wang, Z., Butel, N., Santos-González, J., Borges, F., Yi, J., Martienssen, R. A., Martinez, G., & Köhler, C. (2020). Polymerase IV Plays a Crucial Role in Pollen Development in Capsella. *The Plant Cell*, *32*(4), 950–966. https://doi.org/10.1105/tpc.19.00938
