# Supplementary material for: RNA Pol IV induces antagonistic parent-of-origin effects on Arabidopsis endosperm
Source: PLoS Biol. 2022 Apr 7;20(4):e3001602. doi: 10.1371/journal.pbio.3001602 (PMC9017945; doi:10.1371/journal.pbio.3001602)
Supplement: Attachment — Submitted filename: Satyaki_Response_030222.pdf [file pbio.3001602.s032.pdf]

Response to Reviewers

Satyaki and Gehring

PLoS Biology

Rev. 1:
I could quibble about how functional significant some of these differences are, but the authors have essentially satisfied my concerns.

Response: Thank you for your comment. We agree that functional significance is important, and we believe our findings on the different response of paternal excess crosses to loss of maternal or paternal Pol IV (Figure S1) represent functional significance. However, there remains much to do in this regard.

In order to better communicate the importance of the antagonistic effects of maternal and paternal Pol IV to the reader, we have also slightly modified the presentation of data in Fig 3. We have added one more gene to the bar graphs that illustrate the antagonistic effects (Fig 3C). We have also modified presentation of the scatter plot (Fig 3F). Genes that are antagonistically influenced by maternal and paternal *nrpd1* are colored purple while genes not antagonistically regulated are colored gray. The slope of both sets is indicated in the panel. Finally, we merged Figures 3E/F (from the previous version of the manuscript) into one panel and Figures 3G/H from the previous version into one panel. This allows easy comparisons between relevant datasets (for example, genes upregulated in mat *nrpd1+-* and *nrpd1/-* vs genes upregulated only in mat *nrpd1+-)* . We hope that these modifications will help better convey the magnitude of the antagonistic effects.

Reviewer 2 (below) pointed out that we missed responding to a few of reviewer 1's comments in our original response. We include our response here.

Page 16, line 9. So ideally, one would want to look at a highly polymorphic out-crosser. I'm not suggesting that for this paper, but would this be a good test of the hypothesis? How about maize?
Response: We agree that looking at an outcrosser would be a great next step to test our model. Unfortunately, loss of Pol IV induces severe phenotypes in the outcrossers that have been examined (Grover et al 2018 and Wang et al 2020) to date. We will of course continue to be on the lookout for a suitable system to test this model in.

Page 16, line 15. But the pat and mat heterozygotes looked the same. No differences in phenotype?
Response: We did not find any easily observable seed phenotype.

Page 17, line 22. I'm confused, since aren't most 24 nt smRNAs dependent on POLIV (Figure S2)? Do you mean differentially regulated smRNAs? Expression of POLIV specifically in one or the other parent?
Response: Correct. If we lose one parent's Pol IV, we don't see loss of all sRNAs arising from the chromosomes inherited from that parent.

Page 17, line 23. How are you defining important here? Important because at least in the female NPRD1 is not expressed in the central cell? But not important, since this mutant has no effect on phenotype except in an imbalanced cross.

Response: Important because it has significant effects on gene expression in the endosperm. The changes in gene expression profile suggest potential phenotypes that we can explore in the future.

Page 18, line 13. For this reason, it would be nice to know much more about these genes. Mutant phenotypes, for instance. And how many of these have this feature conserved?

Response: We agree. The seed specific functions of most genes is unclear and knowing more about these genes and their mutant phenotypes are some great next steps.

Rev. 2: Thomas Städler – note that this reviewer has signed the review
This manuscript is a revised version of a manuscript that I previously reviewed for Review Commons. In my mind, it was a solid piece of work back then, and now has been revised based on the extensive comments by the three original referees. Overall, the Results section has been successfully streamlined, and the Discussion section has been expanded to better reflect the implications of this work. There are, however, some remaining issues that I would like to have addressed before publication.

Response: We would like to thank Dr. Städler for his interest in this study and for his valuable comments.

(1) I am curious as to the omission of parts of all reviewers' comments from the "Response to Referees" part of this submission. For example, in my case, only comments that I listed under the sections "Major Comments" and "Minor Comments" have been replicated and addressed by the authors, whereas the required sections "Evidence, reproducibility and clarity" and "Significance" have been left out. Exactly the same applies to the feedback given by referee 3, and some of the minor comments made by referee 2 are "missing". I can understand that my longish and generally very positive assessment previously made in my "Summary" has gone without specific responses, but I did make specific suggestions in the "Significance" part that I would like to have addressed. I could understand why the authors might feel that including my suggestions would make their paper too speculative, but this should be explicitly stated in the "Responses".

Response: We apologize for accidentally omitting responses to some sections of Dr. Städler's review. We address these here (based on context, we believe Dr. Städler was Reviewer 1 for Review Commons, and we paste the additional review information and our responses to the original reviewer 1 below).

"**Summary: from previous round of reviews**

This study addresses key aspects of gene regulation in the developing endosperm of flowering plants. The endosperm is the product of the fusion of the (normally diploid) female central cell with one of the sperm cells, and is indispensable for nourishing the developing embryo, among other important functions. Evolutionary models predict that in flowering plants, the endosperm ought to be the tissue in which parental conflict over the allocation of (female) resources to progeny should manifest. Consequently, endosperm gene expression (including the phenomenon of genomic imprinting) and developmental trajectories have been studied from various perspectives, including the possibility of the fast build-up of reproductive barriers due to failing endosperm (and thus seed) development.

More specifically, this study utilizes knock-out mutants of the NRPD1 gene, which codes for the

largest subunit of RNA Polymerase IV (Pol IV), which is part of the RNA-directed DNA methylation (RdDM) pathway. It builds on previous work by the authors that suggested an important role for Pol IV in mediating allelic dosage in developing endosperm, that small interfering RNAs are produced from both paternally and maternally-derived alleles (Erdmann et al. 2017; contra purported claims by other labs), and that normally inviable seeds from paternal-excess crosses (2n x 4n) can be largely rescued by knocking out individual (paternal) components of the RdDM pathway (Satyaki & Gehring 2019).

Here, Satyaki & Gehring characterize a variety of expression responses in reciprocal heterozygotes, i.e. products of crosses between a homozygous WT and a homozygous nrpd1 mutant (all with the Ler and Col-0 accessions of A. thaliana). The resulting heterozygotes differ in whether the maternal parent (mat nrpd1+/-) or the paternal parent (pat nrpd1+/-) contributed the nrpd1 allele. In addition, mRNA and sRNA expression was also assessed for the WT (+/+) and the homozygous nrpd1 lines (-/-).

Key findings of this work are that the loss of Pol IV in maternal and paternal parents has different consequences for endosperm gene expression, some of which appear to be antagonistic. In other words, the presence of a functioning Pol IV in the mother and father have parent-of-origin effects on the resulting endosperm. Furthermore, one parent's copy of NRPD1 was found to be sufficient for the production of most Pol IV-dependent sRNAs, yet with a fairly small number of mostly non-overlapping loci losing sRNAs upon loss of either maternal or paternal NRPD1. Pol IV activity in the father and mother is shown to have distinct impacts on the endosperm's DNA methylation landscape.

Interestingly, while the proportion of mis-regulated genes seems small in both heterozygotes, it is much more restricted in pat nrpd1+/-. Jointly, the authors' results suggest that paternal and maternal Pol IV are genetically antagonistic and that their effects on endosperm transcription in heterozygotes is established before fertilization.

Response: We thank the reviewer for a clear summary of our current and related relevant research.

Significance:

While part of the earlier data from Erdmann et al. (2017) were re-analyzed in the present study, the vast amount of data are new and concern the expression consequences at the diploid level (2n x 2n crosses), and thus may prove to be more relevant for future comparisons with non-model flowering plants, either for normal intraspecific seed development or (partly) failing crosses between slightly diverged evolutionary lineages. In my view, this study presents a significant advance in understanding the downstream consequences (endosperm mRNA and sRNA expression levels, levels and patterns of DNA methylation) of a perturbed Pol IV expression in both parents or the female vs. male parent. Much of the emphasis in the field has been on paternal-excess crosses, within the larger realm of the "triploid block" or the reproductive barriers between plants of different ploidy (typically diploids x tetraploids in both cross directions).

The fact that the molecular consequences of disabled Pol IV in one or both parents were assessed in balanced crosses (and not interploidy crosses) may allow an easier connection to natural phenomena such as (partial) hybrid seed lethality between closely related lineages of flowering plants, where parent-of-origin effects have emerged in the recent literature, both at the

phenotypic level of endosperm/seed growth and at the molecular level (perturbed imprinting in the endosperm, mRNA and sRNA expression levels, etc.). It thus might prove worthwhile to screen recent papers in Solanum, Mimulus, and Capsella to evaluate the possibility of "connections" between the current data and recent, admittedly more descriptive, findings in diverse taxa that don't offer the same genomic resources as Arabidopsis.

What the current version of this work already does is relating the finding of partly antagonistic influences of pat nrpd1+/- versus mat nrpd1+/- on endosperm mRNA expression to evolutionary models championed by D. Haig ("parental conflict", "kinship theory"). My above suggestions would strengthen such connections and likely would broaden the appeal of this work to scientists with diverse backgrounds outside the core expertise of plant molecular/developmental biologists. In any case, I see the prime scientific audience as the latter group, but I see potential to intrigue people with a more evolutionary background.

Response: We thank the reviewer for placing our work in the appropriate context. We appreciate the reviewer's sentiment here and sincerely hope that this paper appeals to a wide audience, including evolutionary biologists. While we think that our work will provide a primer for studies in other species, we feel that we, ourselves, are not yet ready to make some of these connections. Our experience while working on endosperm sRNAs over several years has been that the endosperm is a complex tissue and our *a priori* hypotheses and models often tend to be wrong (an important feature of Satyaki and Gehring (2019)). We think that in the coming years our work, alongside that of Dr. Städler and other researchers, will provide a larger body of empirical data with which we can draw better conclusions about the role of sRNAs in endosperm gene expression and parental conflict across species. For now, we feel confident enough to draw connections to the WISO model.

(2) Although I specifically requested that all cited references should include volume and page (or article) numbers, as is the rule for all journals that I am familiar with, Satyaki & Gehring have not delivered, despite their assurances. I count 12 references with such problems, and 2 of those are listed with a single author when in reality there are 4 authors (Grossniklaus et al. 1998) and 6 authors (Köhler et al. 2003), respectively. In addition, in most cases genus and species names are not italicized in the References section, plus a few other formatting issues.

Response: We apologize for our omissions. This seems to be linked to our citation software and we have manually fixed them.

Minor Comments
* Page 3, lines 17-21: the explanation of the rationale underlying the kinship model does not appear to be entirely correct. There might be cases where the inclusive fitness of mothers is not optimal under equal provisioning (e.g. if fathers differ in genetic quality and mothers can somehow sense this); the model's rationale is that relatedness of mothers to all of her offspring is identical, while under a polygamous mating system fathers sire offspring that compete for finite maternal resources with their half-sibs.
Response: We agree with the reviewer and have changed the text to *"These parent-of-origin effects on endosperm development have been interpreted under the aegis of the parental conflict or kinship model (Haig, 2013; Haig & Westoby, 1989). According to this model, when a mother mates with more than one father, the inclusive fitness of the mother <u>may be optimized if</u> her resources are equally distributed among her progeny<u>, to which she is equally related</u>. The inclusive fitness of the father is optimal when his progeny are able to acquire more <u>finite</u> maternal resources than other half-siblings.*

* Page 4, 1st paragraph: at first occurrence in the main text, the names of genera should be spelled out (Arabidopsis, Brassica, Capsella).

Response: Thank you for pointing this out. We have made the necessary changes.

* Page 16, 1st paragraph of section "Pol IV, conflict, ….": the beginning of this section needs a few citations, as well as more details. I don't think that all viviparous species would qualify; the relationship asymmetry obtains under multiple paternity or, more generally, scales with the degree of relatedness between (multiple) parents.

Response: We agree. We have changed the sentence to read "*Parental conflict theory predicts that in viviparous, <u>polyandrous</u> species, mother and father have antagonistic..*"

We have also expanded on the detection of antagonistic effects: *"Analogous to observations for cryptic meiotic drive-suppression systems (Lindholm et al., 2016), antagonistic parental effects are likely to be balanced in the individuals within an inbred population…."*

We have also added additional references here: Städler et al, 2021, Florez-Rueda et al, 2021 and Lindholm et al, 2016.

* Page 18, line 7: technically, Brandvain & Haig (2005) called it "weak inbreeder/strong outbreeder" model.
Response: We apologize for this oversight. We have made the necessary change.

* Page 18, lines 6-19: this constitutes the new paragraph that the authors inserted in response to my previous comments. There is nothing "wrong" with it but I think it is omitting some recent developments suggesting that the WISO hypothesis needs to be broadened to include differences in levels of parental conflict among obligate outcrossers. In other words, it now appears that differences in the breeding system are not the only factor influencing levels of parental conflict, as recent empirical data in Mimulus (Coughlan et al. 2020, Curr. Biol.) and Solanum (Roth et al. 2019, Genetics; Städler et al. 2021, Curr. Opin. Plant Biol.) suggest that larger differences in effective population size between hybridizing, outbreeding lineages result in greater asymmetry in seed development and levels of seed failure. These aspects are at the core of what I suggested to consider in my previous referee comments (alluded to above under major point 1).
Response: We thank Dr. Städler for this insightful comment. We have not thought about this in sufficient detail in the past and we will surely consider these angles as we consider future experiments. In this manuscript, we have decided to err on the side of caution and not try to speculate about how Pol IV fits into these truly intriguing dynamics as our understanding of intra and inter-specific variation in Pol IV function is fairly limited.

* Page 20, line 9: substitute "were" for "was" at end of line.
Response: Thank you! We have fixed it.

* Page 22, line 4: there is a duplicate "that" before "2/3".

Response: Thank you! We have fixed it.

* References: the above-mentioned incomplete information concerns Blevins et al. 2015, Gehring et al. 2011, Ji et al. 2014, Kim et al. 2013, Martin 2011, Mi & Thomas 2009, Milbocker &

Sink 1969, Panda et al. 2020, Pignatta et al. 2014, 2015.
Response: We have manually added page numbers for these references.

* Figure S5: the figure legend mentions Schon & Nodine 2017, but the full citation is not given anywhere.

Response: We have remedied this by adding a line to the methods section: *"Seed coat contamination in our samples was ruled out by examining transcriptome data using a previously published (Schon & Nodine, 2017)."*

Rev. 3: Steven E. Jacobsen – note that this reviewer has signed the review
The authors have made all of the recommended changes and the paper is ready for publication.

Response: We thank Dr. Jacobsen for his recommendations. In response to comments from current reviewer 2, we have pasted below original comments from reviewer 3 that we deemed did not require a response.

Reviewer #3 (Evidence, reproducibility and clarity (Required)):

Short Summary:

In this study, Satyaki and Gehring investigate the role of RNA Pol IV in Arabidopsis endosperm, focusing on parent-of-origin-specific functions and potential mechanisms. Using a combination of gene expression, sRNA profiling, and DNA methylation data from reciprocal crosses, they find that maternal loss of Pol IV has distinct, and in some cases opposite, effects on gene expression compared to paternal loss of Pol IV. This is also true to a lesser extent for sRNAs and DNA methylation, consistent with the function of RNA Pol IV in driving 24nt sRNA production and targeted DNA methylation through the RdDM pathway. DNA methylation was more strongly affected by paternal Pol IV loss while expression was much more affected in maternal Pol IV loss. Surprisingly, the authors consistently find no evidence that the minor changes sRNA production or DNA methylation in maternal/paternal nrpd1/+ heterozygotes are correlated with gene expression changes in either heterozygote. However, while the mechanism remains unclear, evidence presented here that maternal and paternal Pol IV can have opposite, additive effects on gene/sRNA expression and phenotype (rescue of paternal excess crosses) is convincing and an interesting finding, potentially consistent with the idea that Pol IV helps mediate parental conflict in endosperm.

Reviewer #3 (Significance (Required)):

Significance:

PolIV is a plant-specific polymerase that functions part of the plant-specific RNA-directed DNA methylation pathway, which has been very well characterized in Arabidopsis. Mutations in PolIV were previously shown to rescue paternal excess crosses when inherited paternally (Erdmann et al. 2017), and this study extended that finding to show that maternal vs. paternal loss of Pol IV has opposite effects on seed survival in paternal excess crosses. Only one other example (met1) of opposed paternal vs. maternal effects on seed development is known, making Pol IV a useful tool for studying why and how these effects occur. As the authors note, the dominant theory on 'why' involves Pol IV mediating parental conflict over resource allocation in the seed,

and the opposite effects of Pol IV maternal/paternal loss at some genes support this hypothesis. The 'how' remains unclear, although this study eliminates several possibilities, and the most likely remaining model is that Pol IV parent-of-origin specific effects occur mostly in trans. Future work can build on these findings to identify the mechanism by which Pol IV achieves these parent-of-origin specific effects.

My background is mostly in plant epigenetics and genomics.

Referee Cross-commenting

The other referee comments seem fair, and I have not comments at this time.
